# Peripheral Blood Lymphocyte Analysis in Oligo- and Polyarticular Juvenile Idiopathic Arthritis Patients Receiving Methotrexate or Adalimumab Therapy: A Cross-Sectional Study

**DOI:** 10.3389/fped.2020.614354

**Published:** 2020-12-10

**Authors:** Arnold Nagy, Bernadett Mosdosi, Diana Simon, Timea Dergez, Timea Berki

**Affiliations:** ^1^Department of Paediatrics, University of Pecs, Medical School, Pecs, Hungary; ^2^Department of Immunology and Biotechnology, University of Pecs, Medical School, Pecs, Hungary; ^3^Institute of Bioanalysis, University of Pecs, Medical School, Pecs, Hungary

**Keywords:** JIA, DMARD, MTX, TNF-alfa inhibitor, lymphocyte populations, infection

## Abstract

Juvenile idiopathic arthritis (JIA) is an umbrella term for seven distinct chronic immune-mediated diseases. Disease-modifying anti-rheumatic drugs (DMARD) are used to treat the underlying joint inflammation as well as extra-articular manifestations. Immunosuppression is a considerable side effect of the drugs. The main goal of this study was to investigate the effect of different JIA therapies on leukocyte subpopulations, which play a role in immune-defense. Three study groups were established. The first group consisted of JIA patients treated with methotrexate solely, the second one received a combination of methotrexate (MTX) and adalimumab (ADA). The control group was made up of the patients' healthy siblings. A total of 63 children were recruited. Fourty-one children with JIA and 22 healthy controls were included in the study. The absolute number of CD3+ T-cells was significantly elevated in patients treated with biological therapy compared to healthy controls (p2 = 0.017). In contrast, the number of CD56+ natural killer cells was significantly lower in children receiving biological therapy in comparison with healthy donors (p2 = 0.039). A significant alteration was also demonstrated between patients treated with MTX and MTX/ADA group concerning CD 19+ B-cells (p3 = 0.042). This is the first study that demonstrates significant alterations in the number of B-cells and T-cells with a relative decrease of NK-cell ratios in JIA patients receiving different DMARD therapy.

**Clinical Trial Registration:** NCT03833271. 21.01.2019.

## Introduction

Juvenile idiopathic arthritis (JIA) is the most common chronic rheumatic disease of unclear etiology and pathophysiology in childhood. The disorder falls into seven distinct categories according to the International League of Associations for Rheumatology (ILAR) (1).

Early, aggressive therapy is crucial to control inflammation and joint destruction (2–5). The remission phase is continued with a firm step down scheme, to prevent toxic adverse events. After initial non-steroid anti-inflammatory drug therapy, conventional treatment is a synthetic disease-modifying anti-rheumatic drug (sDMARD). Methotrexate (MTX) is the most common first-line sDMARD, it is usually administered with intra-articular or systemic steroid. The efficacy and safety of MTX were confirmed in numerous clinical trials (6, 7). However, about 30% of the patients are non-responsive to MTX treatment. Understanding the immunological pathways and mechanisms related to the pathogenesis of JIA led to novel targeted therapy. Blocking TNF-alfa molecule in the inflammatory cascade triggers remission, better disease control and cessation of the clinical symptoms. Anti-TNF-alfa agents used in the treatment of JIA are infliximab (INX), adalimumab (ADA), certolizumab pegol, golimumab, and etanercept (ETA). These drugs are confirmed to be extremely efficacious in numerous trials (8–11). However, their immunosuppressive effect has to be taken into account.

The relationship between rheumatoid arthritis (RA), or the treatment of the RA and the adverse effects are demonstrated in various trials (12–15). Yet, the association between JIA itself, its therapy and their immunosuppressive effect leading to infection is not clear.

Besides novel findings, there is still little data available regarding the alteration of immune cells which control infection. A comprehensive review presented by Swart et al. found that the risk of developing an infection is a consequence of JIA itself rather than the therapies' (16). It is in line with the investigations performed on different medications. MTX did not increase the risk of infection in RA patients (6, 17–19). The safety studies scrutinizing anti-TNF-alfa drugs failed to show a significant difference between the JIA patients treated with or without these drugs (8, 9, 20–22).

The primary aim of this study was to evaluate the influence of different treatment modalities on the composition of lymphoid cells in JIA patients.

## Materials and Methods

It is a single-center prospective study from the Department of Pediatrics, University of Pécs Clinical Center. Fourty-one children with JIA and 22 healthy controls were enrolled in the analysis: 15 (23.8%) patients treated with a stable dose of MTX (15 mg/m^2^/week per oral). The other treatment group (*n* = 26, 41.2%) received MTX and ADA (Humira, AbbVie^®^). ADA's dose was 20 mg (under 30 kg) or 40 mg every second week subcutaneously. Only patients on remission were selected to rule out the influence of disease activity on peripheral blood (PB) cells. The definition of inactive disease was in congruence with the paper of Wallace (23). Exclusion criteria were active arthritis, ongoing acute illness, the five other subtypes of JIA or a DMARD therapy apart from MTX.

The patients arrived on the day of the investigation at our Clinic's Allergy and Immunology Outpatient Care Unit. After their clinical evaluation (including measurement of body weight, height and vital signs) a detailed rheumatologic investigation was performed on each of them. Peripheral venous blood samples were taken from all participants. Routine laboratory tests encompassed complete blood count, erythrocyte sedimentation rate (ESR), C-reactive protein (CRP), immunoglobulins (IgA, E, G, M) and complements (C3, C4, CH50-total complement). The immune serologic test comprised of anti-nuclear antibody (ANA), cyclic citrullinated peptide (CCP) and rheumatoid factor (RF) antibodies analysis. Peripheral blood lymphocytes were analyzed by flow-cytometry. 50 μl of peripheral blood was used for the immunofluorescent staining with the following antibodies against CD3, CD4, CD8, CD56, CD45, CD19, CD5, CD27 IgD, CD25, HLA-DR, CD45-RA, CD45-RO, (Beckton Dickson and Company Biosciences, San Jose) for 30 min at RT. Subsequently, 2 ml lysis buffer was added to the cells and incubated at room temperature for 10 min to eliminate the red blood cells. After that the cells were washed in PBS/BSA/azide and centrifuged at 1,000 rpm for 5 min. The supernatant was then removed and the cells were fixed with 300 μl FACS-FIX solution. The samples were kept at 4°C until the measurement was taken. Flow-cytometric detection was performed on a FACSCalibur™ Flow Cytometer (BD Biosciences) and the results were analyzed using the CellQuest Pro 5.1 (BD Biosciences) software. 10^5^ cells were collected from the lymphocyte gate and the lymphocyte subpopulations were analyzed and expressed as percentages of total lymphocytes. The following cell types were investigated: CD56+ natural killer cells (NK), CD3+CD56+ natural killer T cells (NKT), CD3+CD8+ cytotoxic- and CD3+CD4+ helper T lymphocytes, CD19+CD5+ B1- and CD19+CD5- B2 B lymphocytes, CD4+CD25high+ regulatory T and CD3+CD25 medium+ activated T cells, CD3+HLA-DR+ activated T cells, CD8+HLA-DR+ activated T cytotoxic cell, CD3+CD45RA+ naive and CD3+CD45RO+ memory T cells, CD19+IgD+CD27- naïve B cells, CD19+IgD+CD27+ non-switched B and CD19+CD27+IgD- switched B cells.

The study was performed according to the Declaration of Helsinki and the protocol was approved by the Local Ethics Committee (SN: 7387, University of Pecs, Medical School). All informed content was obtained either from JIA patients, healthy controls or from their parents.

### Data Analysis and Statistics

Statistical analysis tests were performed using IBM SPSS Statistics 25 Software. A descriptive statistical analysis was performed. Continuous variables were expressed as median and interquartile range or mean ± standard deviation if adjusted to a normal distribution, and evaluated by Kolgomorov-Smirnov tests when appropriate. The main primary outcome of the analysis was to compare immune-cells and laboratory parameters in case of the 3 groups. For quantitative variables, the Mann-Whitney test or two-sided Student's *t*-test were used. If the variance was not homogenous the Welch test was applied, in case of homogeneity of variances, ANOVA was applied. The categorical data were analyzed using contingency tables and the chi-squared or Fischer's test, as appropriate. Statistical significance was established as a *p*-value of < 0.05. P1 indicates the comparison between MTX group and healthy controls, p2 reveals the relationship among MTX/ADA group and controls and p3 is the sigma of MTX and MTX/ADA groups.

## Results

The demographic data and clinical characteristics of the participants are listed in Table 1. Fourty-one JIA patients and 22 healthy siblings took part in the study. We focused on two distinct, but relatively homogeneous subsets of patients with JIA in remission: 25 (61%) children with oligoarthritis (OA) and 16 (39%) with polyarthritis (PA). Twelve (48%) have persistent and 13 (52%) have extended OA. Fifteen (36.5%) patients (12 persistent OA, 3 PA) treated with MTX alone and 26 (63.5%) children were on a stabile combination of MTX and ADA, (50% extended OA, 50% PA) precisely. The patients treated with MTX/ADA was on a stable dose of MTX therapy for 1.92 (0.66–3.42) years before the initiation of ADA. PA patients had significantly longer disease duration (*p* = 0.005) comparing with OA patients. Also, they had been on therapy significantly for a longer period (mean R = 18.18 and 25.41, *p* = 0.049).

**Table 1:** Baseline characteristics of the study population.

	**JIA patients treated with MTX** **(*n* = 15)**	**JIA patients treated with MTX/ADA (*n* = 26)**	**Healthy controls (*n* = 22)**
Age, mean ± SD (years)	7.12 ± 4.81	7.47 ± 4.37	12.4 0± 4.02
Males, no. (%)	5 (33.3)	12 (46.15)	13 (59.09)
Median duration of JIA, years (range)	2.00 (0.25–13.83)	3.80 (0.91–11)	n.a.
Median duration of the start of therapy, years (range)	2.00 (0.25–13.83)	1.88 (0.25–7.58)	n.a.
Systemic steroid, no. (%)	7 (46.6)	15 (57.69)	n.a.
JIA subtype • oligo JIA, no. (%) • poly JIA, no. (%)	12 (80) 3 (20)	13 (50) 13 (50)	n.a.

At the beginning of the disease course, seven MTX patients and 17 MTX/ADA patients received intraarticular steroid injection. Moreover, seven on MTX and 15 on MTX/ADA got systemic glucocorticoid (GC) because of the severity of the disease. However, at the time of investigation GC was completely tapered down with a minimum of 4 months before the study. Uveitis was not observed in our patients. Regarding autoantibodies seven (17%) patients had ANA-positivity, one (2.4%) had anti-CCP and eight (19.5%) had RF-antibody. There were no significant alterations observed among the three groups concerning acute or chronic inflammatory laboratory parameters and complete blood count. Common laboratory values are demonstrated in Table 2.

**Table 2A:** Basic laboratory findings.

	**JIA patients treated with MTX**	**JIA patients treated with MTX/ADA**	**Healthy controls**	**Significance (*p-*value)**
ESR *(mm/hour)*	12.00 ± 7.82	10.85 ± 6.63	8.68 ± 5.88	0.295 0.509 0.851
CRP *(mg/l)*	0.85 ± 1.67	1.03 ± 2.13	1.09 ± 1.68	0.923 0.992 0.957
Leukocyte *(G/l)*	6.92 ± 2.38	7.22 ± 1.47	6.77 ± 1.35	0.959 0.630 0.846
ANC *(G/l)*	3.94 ± 1.57	3.71 ± 1.03	3.69 ± 1.14	0.803 0.999 0.814
Monocyte *(%)*	0.33 ± 0.18	0.38 ± 0.12	0.33 ± 0.84	0.988 0.429 0.392
Thrombocyte *(G/l)*	304.06 ± 62.47	295.04± 58.78	302.05 ± 89.23	0.996 0.939 0.917

*Data aremeans ± standard deviation. p1 = MTX and control, p2 = MTX/ADA and control, p3 = MTX and MTX/ADA*.

**Table 2B:** Basic laboratory findings.

	**JIA patients treated with MTX**	**JIA patients treated with MTX/ADA**	**Healthy controls**	**Significance (*p-*value)**
Immunoglobulin G *(g/l)*	11.33 ± 2.68	11.42 ± 2.14	11.13 ± 1.91	0.962 0.896 0.991
Immunoglobulin A *(g/l)*	1.77 ± 0.98	1.65 ± 1.01	1.49 ± 0.83	0.659 0.840 0.916
Immunoglobulin M *(g/l)*	1.24 ± 0.73	1.38 ± 0.58	1.14 ± 0.73	0.903 0.454 0.802
C3 *(g/l)*	1.24 ± 0.22	1.22 ± 0.13	1.30 ± 0.22	0.492 0.242 0.951
C4 *(g/l)*	0.23 ± 0.08	0.22 ± 0.06	0.23 ± 0.08	0.995 0.930 0.969
CH50 *(U/ml)*	51.30 ± 24.85	50.44 ± 17.15	66.28 ± 30.52	0.189 0.091 0.994

*Data are means ± standard deviation. p1 = MTX and control, p2 = MTX/ADA and control, p3 = MTX and MTX/ADA*.

Concerning the absolute numbers of CD3+ T-cells, a significant difference was demonstrated between MTX/ADA and healthy control groups (2067.07 ± 642.04 (95% CI: 1807.74–2326.40) and 1628.36 ± 353.42 (95% CI: 1471.66–1785.06), p2 = 0.017). CD4+ T-helper and CD8+ cytotoxic cells showed a remarkable increase in the MTX/ADA group comparing controls, however, it did not reach a level of significance (p2 = 0.054 and p2 = 0.060). Our investigation extended to other T-cell subsets as CD3/CD25, CD4/25, CD4/25/FOXP3, DR/CD3, DR/CD8, CD3/45RA, CD4/45 RO, NKT-cells which did not show a significant difference between groups (complete data set available on request).

The absolute number of CD56+, natural killer (NK)-cells has decreased significantly in the MTX/ADA group comparing healthy controls (254.70 ± 131.25 (95% CI: 182.02–327.39) and 341.50 ± 152.48 (95% CI: 273.89–409.10) p2 = 0.039).

An alteration in the humoral compartment was also observed. A significant change was demonstrated between MTX and MTX/ADA patients in the case of total CD19+ naïve B-lymphocyte number (199.60 ± 94.95 (95% CI: 147.01–252.18) and 291.30 ± 126.69 (95% CI: 240.13–342.48) p3 = 0.042). We also investigated whether this change had a functional consequence, therefore we assessed immunoglobulin (IgM, IgG, and IgA) levels in the patients' sera. The age-matched Ig levels were in the normal range. Furthermore, no difference was presented regarding pre-switched and switched memory B-cells and B-cells bearing the CD5 marker between the three groups.

**Figure 1:**
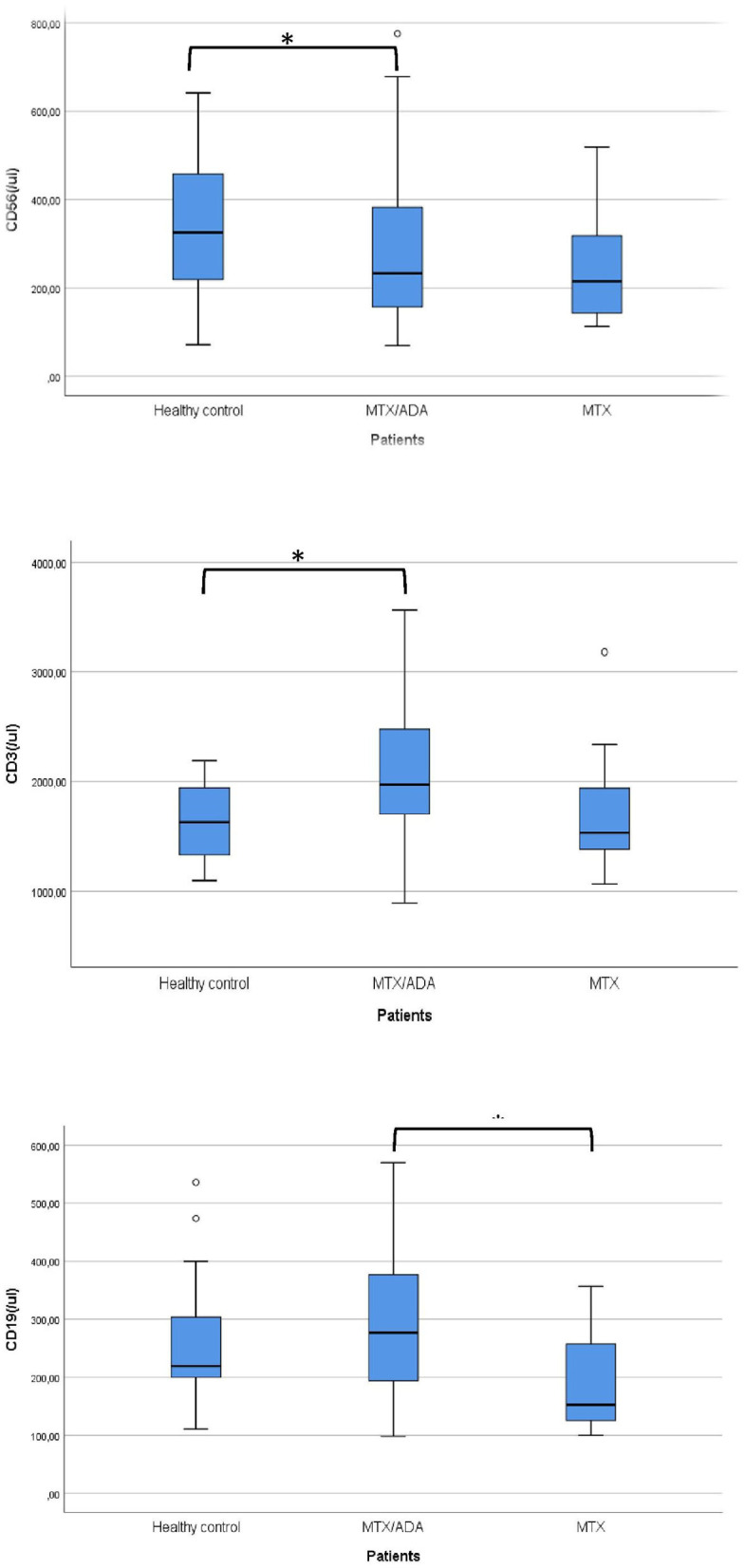
Significant alterations are demonstrated between the investigated groups. *Means significant alteration and °means outlier.

Complements (C3, C4, total complement-CH50) were also analyzed, but no alteration was found among the investigated groups. Table 3 and Figure 1 show the distribution (%) of lymphocytes and the absolute cell numbers analyzed with flow cytometry.

**Table 3:** Flow-cytometry.

	**JIA patients treated with MTX** **(*n =* 15)**	**JIA patients treated with MTX/ADA** **(*n =* 26)**	**Healthy controls** **(*n =* 22)**	**Significance (*p-value*)**
**Distribution of lymphocytes**				
Total lymphocytes *(G/l)*	2.23 ± 0.59	2.72 ± 0.78	2.30 ± 0.47	0.933 0.077 0.057
CD3 *(%)*	75.36 ± 5.74	75.90 ± 7.23	70.57 ± 6.15	0.081 **0.018** 0.966
CD3/CD4 *(%)*	42.18 ± 6.18	42.54 ± 9.36	39.61 ± 7.31	0.606 0.421 0.989
CD3/CD8 (%)	27.95 ± 6.63	27.69 ± 6.42	25.38 ± 5.77	0.442 0.415 0.991
CD19 (%)	8.83 ± 3.17	10.66 ± 3.67	11.38 ± 3.42	0.082 0.754 0.246
Preswitched (naïve) B *(%)*	78.48 ± 7.73	79.90 ± 7.49	78.99 ± 8.20	0.979 0.918 0.841
Switched B *(%)*	11.04 ± 6.03	11.54 ± 7.09	12.26 ± 5.10	0.833 0.918 0.967
CD4/25^hi^ *(%)*	2.03 ± 1.55	2.00 ± 1.18	2.66 ± 1.81	0.486^*^
CD3/45RA *(%)*	43.21 ± 11.72	43.31 ± 11.35	39.01 ± 10.79	0.509 0.390 1.000
CD3/45RO *(%)*	32.27 ± 9.08	32.95 ± 10.16	31.85 ± 8.75	0.990 0.914 0.973
CD56 *(%)*	12.01 ± 6.13	10.73 ± 6.10	14.96 ± 6.33	0.187 **0.014** 0.408
**Absolute number of lymphocytes**				
Total lymphocytes *(/ul)*	2231.33 ± 599.72	2724.61 ± 784.63	2308.63 ± 476.85	0.933 0.077 0.057
CD3 *(/ul)*	1697.46 ± 548.63	2067.07 ± 642.04	1628.36 ± 353.42	0.921 **0.017** 0.092
CD3/CD4 *(/ul)*	958.66 ± 379.10	1108.80 ± 240.30	915.09 ± 251.85	0.890 0.054 0.0237
CD3/CD8 *(/ul)*	628.33 ± 229.17	788.03 ± 400.47	585.36 ± 180.47	0.905 0.060 0.238
CD19 *(/ul)*	199.60 ± 94.95	291.30 ± 126.69	266.31 ± 110.11	0.197 0.731 **0.042**
CD56 *(/ul)*	296.89 ± 187.15	254.70 ± 131.25	341.50 ± 152.48	0.175 **0.039** 0.492

Data are means ± standard deviation. p1 = MTX and control, p2 = MTX/ADA and control, p3 = MTX and MTX/ADA.

* *Non-normal distribution. Bold values indicate significant alterations*.

## Discussion

Treatment with different DMARD therapies lead to an improvement in JIA patients. Infection is one of the most uncertain adverse effect of these drugs.

JIA patients might have an increased risk of infections on the basis of the disorder. The autoimmune or inflammatory process of the disease is associated with immune dysregulation. Therefore, the disease course might have an influence on developing infection regardless of therapy (24–26). However, the immunosuppressive therapies could furthermore elevate the risk. A recently published meta-analysis revealed important findings about the number of infections observed in JIA patients treated with TNF alpha inhibitors. The study reported that the majority of infections were located in the upper respiratory tract. Yet, significant increase in the infection rate could not be detected (27). Beukelman et al. reported that steroid-sparing DMARD therapies may reduce the risk of severe infections in children since high-dose steroid therapy may increase the infection rate 3-fold in JIA patients (25, 26).

For all we know, this is the first study in pediatric population which compares lymphocyte subpopulations in healthy individuals and JIA patients in remission receiving different DMARD therapy.

T-cells play a crucial role in JIA pathogenesis. A recently published report which investigated active, non-treated JIA patients, demonstrated significantly higher levels of CD4+ and CD8+ T-cells, all along with total lymphocyte count (28). Conversely, lower numbers of CD8 cells were observed in a cohort of patients in remission without medication (29). There are a few reports about T-cell subset changes during DMARD therapies. In the study of Bulatovic the enhancement of effector T cell function was observed in JIA patient treated with MTX (30).

In our study elevated absolute numbers of CD3+, CD4+, CD8+ T-lymphocytes in the TNF-alfa inhibitor cohort were revealed, compared to MTX group or healthy controls. The count of CD3+ T-cells was significantly increased. There was no alteration between MTX treated patients and healthy controls. The accumulation of effector T-cells in PB could indicate a more destructive disease with aggressive pro-inflammatory immune response in the TNF-alfa group. Consequently, this could be the reason why these children receive anti-TNF-alfa therapy to reach an inactive disease state.

Several studies have investigated the alterations in peripheral B cell subsets in JIA patients (31–33). B-lymphocytes also have a critical role in immune response, they act as antigen-presenting cells and they are responsible for the production of auto-antibodies. Previous studies showed that naive B lymphocytes were significantly increased in JIA and RA patients, than in healthy controls (28, 34, 35). A study by Glaesener et al. revealed a direct reducing effect of MTX on early B-cell development (36). In line with the previous study, our results also revealed a non-significant decrease CD19 level observed in our patients receiving MTX. Still, it did not have any functional consequences, since age-matched Ig levels were in the normal range in all three groups.

The switched memory B cells are the most widely presented subset of cells in the synovial fluid. They produce ANA and their levels are elevated in patients with early disease onset. In OA and PA JIA patients, an increased number of switched memory B-cells was reported with the correlation of disease activity (31). They demonstrated that in remission phase anti-TNF-alfa agents inhibit the elevation of these cells both in PB and on the site of inflammation. Our investigation did not find any alteration concerning pre-switched and switched memory B-cells in either group. Therefore, it should be emphasized that no suppression of humoral immunity were observed.

To date, our results demonstrated for the first time that CD56 NK-cells were significantly decreased in the MTX/ADA group compared to healthy controls. It might be an explanation of the increased number of mild, viral, upper respiratory tract infections demonstrated in various trials (27). Other parts of the innate immune-system like complements and phagocyte levels did not show any alteration.

The present study has some limitations. Only the quantitative assessment of the circulating lymphocytes was carried out, the qualitative measurement through cytokine levels is lacking. Parallel investigation of the synovial fluid is also missing as our patients were in an inactive disease state, therefore performing an articular puncture was not possible. Cross-sectional study desing is also a limitation of the study.

Longitudinal follow-up is necessary to analyse the alteration in cell levels, also these cells should be investigated in patients off medication.

## Conclusions

We suggest that the quantitative alterations found in the MTX/ADA group could be a result of the more aggressive disease course, thus do not simply belong to anti-TNF-alfa therapy. We have to emphasize that ADA's therapeutic effect largely overgrows its adverse effects. MTX is a good choice of therapy concerning the cost-benefit ratio.

## Data Availability Statement

The raw data supporting the conclusions of this article will be made available by the authors, without undue reservation.

## Ethics Statement

The studies involving human participants were reviewed and approved by The Ethics Committee of The University of Pecs (SN: 7387, University of Pecs, Medical School). Written informed consent to participate in this study was provided by the participants' legal guardian/next of kin.

## Author Contributions

Conceptualization: AN and BM. Data collection: AN and Adam Gyori. Methodology: TB, DS, and BM. Statistics: TD. Writing—original draft preparation: AN. Writing—reviewing and editing: BM, TB, and AN. Supervision: BM and TB. All authors fulfill the criteria for authorship. Every author participated in reviewing the manuscript, has seen the submitted version of the paper and approved the submission.

## Conflict of Interest

The authors declare that the research was conducted in the absence of any commercial or financial relationships that could be construed as a potential conflict of interest.

## Abbreviations

ADA: adalimumab
ETA: etanercept
CD: cluster of differentiation
INX: infliximab
JIA: juvenile idiopathic arthritis
MTX: methotrexate
OA: oligoarticular
PA: poly-articular
PB: peripheral blood
RA: rheumatoid arthritis
sDMARD: synthetic disease-modifying anti-rheumatic drug
TNF-alfa: Tumor Necrosis Factor Alpha.
